# UAV image-derived canopy traits for predicting alfalfa fall dormancy and forage yield in Mediterranean environments

**DOI:** 10.3389/fpls.2026.1841672

**Published:** 2026-07-17

**Authors:** Francisco González, Hamza Armghan Noushahi, Danae Hernández, Miguel Garriga, Alejandro del Pozo, Macarena Gerding, Claudia Muñoz-Espinoza, Salvador A. Gezan, Luis Inostroza

**Affiliations:** 1 Facultad de Agronomía, Universidad de Concepción, Concepción, Chile; 2 Centro de Mejoramiento Genético y Fenómica Vegetal, Facultad de Ciencias Agrarias, Universidad de Talca, Talca, Chile; 3 VSN International, Hemel Hempstead, United Kingdom; 4 Instituto de Investigaciones Agropecuarias-INIA, Chillán, Chile

**Keywords:** alfalfa breeding, fall dormancy, high-throughput phenotyping (HTP), machine learning, RGB vegetation indices, UAV-based phenotyping

## Abstract

Fall dormancy (FD) and forage yield (FY) are two key traits in alfalfa (*Medicago sativa* L.) breeding programs. However, genetic progress has remained limited over the past decades, largely due to the complexity of alfalfa breeding and the reliance on labor-intensive phenotyping methods. High-throughput phenotyping (HTP) using unmanned aerial vehicles (UAVs) represents a promising alternative for rapid and non-destructive crop evaluation. The objectives of this study were to i) estimate FD using UAV-derived canopy height and RGB vegetation indices and ii) evaluate the predictive performance of machine learning (ML) models for FY estimation. A total of 210 alfalfa populations with diverse genetic backgrounds were evaluated over two growing seasons (2023 to 2025) across seven harvests under Mediterranean conditions in central Chile. FY was measured manually, while FD was estimated using both manual and UAV-based approaches. A total of 19 RGB-derived indices (VIs) including plant height (PH) were extracted and used as predictor variables. Five complex predictive ML models were evaluated: PLS, PCR, SVM, ANN, and MLR. The results showed that UAV-derived FD was significantly correlated with FD obtained through conventional methods (*R*^2^ = 0.88). The automated UAV-based FD phenotyping framework demonstrated slightly higher precision (*R*^2^ = 0.92) and broad-sense heritability (*H*^2^ = 0.69) compared to manual measurements (*R*^2^ = 0.87–0.89; *H*^2^ = 0.64), providing a more reliable selection tool for breeders. Among the tested ML models, SVM and ANN achieved the highest accuracy (*R*^2^ ≈ 0.73) for FY prediction. These findings demonstrate that integrating low-cost RGB imagery with complex modeling offers a promising avenue that could assist in refining future selection strategies for this genetically complex species.

## 1 Introduction

Renowned as the “queen of forages”, alfalfa (*Medicago sativa* L.) possesses outstanding agronomic performance with high forage yield (FY) and serves as an exceptional source of protein and digestible fiber for livestock (Acharya et al., 2020; Noushahi and del Pozo, 2026). It is the most important perennial forage legume worldwide, cultivated on more than 30 million hectares globally (Bouton, 2012). Its prominence in global agriculture is driven by its high year-round biomass production, exceptional nutritional quality, and substantial ecological benefits, including symbiotic nitrogen fixation, carbon sequestration, and soil stabilization (He et al., 2025; Duan et al., 2026). However, the most important challenge facing the industry is the low accumulated genetic gain of alfalfa for FY, estimated at only 10% to 15% over the past 50 years since the late 1970s (Li and Brummer, 2012; Annicchiarico, 2021; Annicchiarico and Pecetti, 2021). In contrast, major annual crops such as maize have achieved consistent yield increases of 1% to 2% per year through hybrid technologies.

The slow breeding progress is attributed to a historical focus on defensive traits and the inherent biological complexity of the species. Moreover, it is an autotetraploid species with an obligate outcrossing reproductive system and exhibits pronounced inbreeding depression (Li and Brummer, 2009; Bouton, 2012). These factors, combined with its perennial growth habit that requires multiyear and multiharvest evaluations, extend breeding cycles to approximately 10 to 14 years (Bouton, 2012). In addition, the broad-sense heritability (*H*^2^) for FY remains relatively low (ranging from 0.15 to 0.30), which limits the efficiency of traditional phenotyping (Inostroza et al., 2021; del Pozo et al., 2023; Noushahi et al., 2025a).

In the context of ongoing efforts to enhance long-term alfalfa FY trends, high-throughput phenotyping (HTP) offers a potential pathway to optimize selection efficiency and phenotypic accuracy, which could support the broader goal of accelerating genetic gain (Inostroza et al., 2016; Biswas et al., 2021; del Pozo et al., 2023; Noushahi et al., 2025a). Unmanned aerial vehicles (UAVs) equipped with multispectral, hyperspectral, thermal, and RGB sensors allow for the rapid, non-destructive estimation of FY across thousands of breeding plots (Lussem et al., 2018; Feng et al., 2020; Kim et al., 2021; Tang et al., 2021). These platforms capture spectral vegetation indices (VIs), which show strong positive correlations with biomass accumulation (Feng et al., 2020). Notably, *H*^2^ for these phenomics traits is often higher than FY. For example, the median heritability of the Green Normalized Difference Vegetation Index (GNDVI) has been reported at 0.64 compared with 0.31 for measured FY (Thapa et al., 2024). Similarly, Noushahi et al. (2025a) reported *H*^2^ values of 0.35 for the RGB-derived HUE index and 0.60 for both b* and v*, whereas FY exhibited a much lower *H*^2^ of 0.16.

Alfalfa productivity and persistence depend on multiple traits. Among these traits, fall dormancy (FD) is a critical determinant of alfalfa adaptation and productivity (Noushahi and del Pozo, 2026). FD is a physiological response triggered by shortening photoperiods and declining temperatures that result in reduced fall regrowth (Liu et al., 2019; Djaman et al., 2021). Alfalfa cultivars are classified into 11 FD categories, which are broadly organized into three groups: i) fall-dormant types (FDT) (FD 1 to 4), ii) semi-dormant types (SDT) (FD 5 to 7), and iii) non-dormant types (NDT) (FD 8 to 11) (Ariss and Vandemark, 2007). FDT cultivars exhibit early dormancy, reduced fall growth, and high winter hardiness, ensuring persistence in higher-latitude environments. NDT cultivars continue growing late into the fall, reach greater plant height (PH), and generally exhibit lower winter survival, leading to higher total annual yield due to their extended growing season (Cunningham et al., 2001).

FD is usually measured using standard tests established by the International Union for the Protection of New Varieties of Plants (UPOV), which involve clipping plots in the fall equinox and manually measuring the PH of the subsequent regrowth approximately 25 to 30 days later (Frangipane et al., 2021). Since regrowth height after fall clipping (winter regrowth) is the primary phenotypic indicator for FD classification, UAV-mounted cameras provide a powerful tool for automating this assessment. Particularly, photogrammetric algorithms such as Structure from Motion (SfM) enable 3D canopy reconstruction by processing overlapping 2D images to generate 3D point clouds and digital elevation models (DEMs). Recent evidence in alfalfa supports a strong correlation between UAV-derived canopy height and FY (Cazenave et al., 2019; Feng et al., 2020; Tang et al., 2021). To date, this HTP approach has not been applied to the estimation of FD categories.

Furthermore, the relationship between high-dimensional phenomics traits and FY is frequently complex. This necessitates a shift from conventional linear models to advanced machine learning (ML) approaches (Xing et al., 2018; Feng et al., 2020). ML approaches, such as partial least squares regression (PLS), support vector machine regression (SVM), and artificial neural networks (ANN), have demonstrated superior performance in handling the complex interactions between spectral, structural, and environmental data (Medina et al., 2024; Yang et al., 2024). In alfalfa, ensemble learning models have achieved coefficients of determination (*R*^2^) of 0.87 for in-season FY prediction (Feng et al., 2020). Furthermore, deep learning models have been developed to integrate environmental factors that achieve high prediction accuracies for growth dynamics parameters (*R*^2^ > 0.95) (Yang et al., 2024). These advanced modeling frameworks enable breeders to prioritize elite genotypes earlier in the season and achieve greater precision than manual methods (Biswas et al., 2021).

It was hypothesized that UAV-derived spectral indices enable accurate FD estimation and FY prediction, with improved performance through complex ML models. The objectives of this study were to i) develop UAV-based HTP methods to estimate FD and ii) evaluate complex models for FY prediction. Phenotypic variation for these traits across 210 alfalfa populations was quantified by integrating UAV imagery with conventional, ground-based methodologies.

## 2 Materials and methods

### 2.1 Plant materials and experimental conditions

A set of 210 alfalfa populations, comprising 185 half-sib progenies and 25 parent populations, was sown in germination trays. Parent selection and half-sib progeny development were performed in previous works by the alfalfa breeding program of INIA Chile (Humphries et al., 2020; Inostroza et al., 2021; Noushahi et al., 2025a). The germination trays were filled with peat moss as the substrate (Kekkila, Finland). The substrate was irrigated daily and fertilized periodically using a solution of 1.1 g L^−1^ Phostrogen (Bayer, UK). At the stage of three fully expanded leaves, seedlings were inoculated with a *Sinorhizobium meliloti* (strain WSM2141) suspension. One week prior to transplanting, seedlings were transferred to a shelter to undergo a hardening process. The 210 alfalfa populations were transplanted at the Santa Rosa Research Station of INIA Chile, situated near Chillán City, Chile (36°31′ S, 71°54′ W). Transplanting was carried out during the spring (November) 2023. The experimental site is characterized by a Mediterranean climate with a historical annual rainfall of 1,100 mm (Noushahi and del Pozo, 2026). The experiment was arranged in an α-lattice experimental design with three replications. Each replicate consisted of 15 incomplete blocks (IBs), each containing 14 populations. The entire experimental field contained a total of 630 plots. Each experimental plot comprised four rows of 1 m in length with adjacent rows spaced 20 cm apart. On each row, seedlings were distributed every 10 cm (40 plants per plot).

### 2.2 Experimental soil characteristics

The soil was classified as thermic Humic Haploxerands and exhibited specific characteristics (Stolpe, 2006). It was measured to have a pH of 6.2 (measured in a water solution at a ratio of 1:2.5, within the 0–20 cm depth). In the top 20 cm, the soil contained 8.0% organic matter, with available nitrogen (N), phosphorus (P), and potassium (K) contents of 7.0 mg kg^−1^, 26.7 mg kg^−1^, and 548 mg kg^−1^, respectively. Prior to transplanting, the soil was adequately prepared by employing a chisel plow and disc harrows. For nutrient enrichment, each plot was supplemented with specific fertilizers including 220 kg ha^−1^ of triple superphosphate (20% P), 30 kg ha^−1^ of boronatrocalcite (11% B), and 100 kg ha^−1^ of zinc sulfate (11% of S and 22% Zn). Experimental plots were irrigated from spring to autumn every 15 days, by a sprinkler system. Manual and chemical weed control were periodically performed in order to maintain experimental plots and alleyways free of weeds.

### 2.3 Forage yield evaluation

FY was evaluated across 630 plots by harvesting at 5 cm aboveground level using a handheld shrub trimmer (Hl75-Ka85, Stihl). After determining the total fresh weight of each plot, approximately 200- to 300-g sample was collected and placed in a paper bag. Both the total plot weight and the sample weight were recorded in the field using a customized system integrating barcodes, a scale, and a computer system. The harvest was completed in a single day by a team consisting of two machine operators and 15 workers responsible for sample bagging. Samples were oven-dried with forced air ventilation at 65 °C until constant weight was achieved for dry matter determination. Two and five cuts were obtained during the establishment (2023/2024) and the 2024/2025 growing seasons, respectively. The dates of the seven cuts were 13 March 2024, 15 May 2024, 25 October 2024, 3 December 2024, 7 January 2025, 12 February 2025, and 19 March 2025.

### 2.4 Fall dormancy estimation

During the autumn season of 2025, FD was determined according to the methodology outlined by the International Union for Protection of New Varieties of Plants (UPOV; TG/6/5). Briefly, the 630 plots were cut 2 days before the autumn equinox (19 March 2025), and PH was measured using a ruler with five measurements per plot at 25, 40, and 60 days after the autumn equinox (21 March 2025). Eight alfalfa accessions with known FD values were used as check cultivars. The accessions included APG45675 (FD = 3), APG6567 (FD = 4), Venus (FD = 5), StaminaGT6 (FD = 6), SARDI7 (FD = 7), APG40234 (FD = 8), WL903 (FD = 9), and SARDI10 (FD = 10). A linear relationship between FD and PH was established using a linear regression analysis in R. The resulting equation (PH = *a* + *b* × FD) was then used to estimate FD for the alfalfa accessions (FD = [(PH − *a*)/*b*]).

### 2.5 UAV-derived phenotypic characterization

The UAV flight was conducted 1 day prior to each cut and 1 h before PH measurement during the autumn season (25, 40, and 60 days after the autumn equinox). The DJI Mavic 3T UAV (DJI, Shenzhen, China) was used for image acquisition. The UAV was equipped with two cameras: the first, a 1/2″ CMOS sensor that allows to capture 48MP RGB photos, and the second, a radiometric thermal camera with a resolution of 640 × 512 pixels.

The flight parameters were set up within the DJI Pilot 2 software installed in the drone remote controller. The photographs capturing parameters comprised 20 m altitude, 80% overlap in both side and heading directions, and a velocity of 2 ms^−1^. The Mavic 3T is equipped with a real-time kinematic (RTK) module to ensure higher accuracy and reliability in positioning. The drone was paired with a DJI D-RTK2 GNSS receiver which is capable of providing centimeter-level accuracy in aircraft positioning during flight. To enhance the accuracy of the location of the experimental plots, eight ground control points (GCPs) were established by positioning targets around and within the experimental area. The DJI D-RTK2 GNSS receiver was used to determine the coordinates of these points.

RGB images were processed using Agisoft Metashape Professional software version 2.1.3 (Agisoft LLC, St. Petersburg, Russia), following the workflow for image processing. This included alignment, alignment optimization, importation and georeferencing of GCPs, generation of point cloud, generation of the DEM based on point cloud, and orthorectification based on DEM. Finally, the DEM and orthomosaics were exported in Tiff format to perform further image analyses.

PH and 19 RGB-derived indices were obtained from orthomosaics and DEM using the image analysis pipeline of the FIELDimageR software (Matias et al., 2020). In brief, 19 RGB-derived VIs were calculated with the fieldIndex function (**Supplementary Table 1**). Image segmentation was performed based on fieldMask function, which allowed to eliminate soil pixels. The HUE index was used as classification criteria (threshold = 0.00). Plot-level information (RGB indices) was extracted through drawing rectangular polygons on each plot with the functions fieldShape and fieldInfo. PH was estimated through the difference between DEM_t2_ − DEM_t1_, where DEM_t1_ and DEM_t2_ are the DEM with bare soil and DEM including the plot elevations, respectively. The DEM_t1_ was estimated with fieldInterpolate function considering 100 points homogeneously distributed across the experimental area. The difference between DEM_t2_ − DEM_t1_ was performed with the fieldHeight function. PH from each plot was extracted with the functions fieldShape and fieldInfo. The DEM image includes only one band (elevation values). Soil pixels were eliminated using random forest classification analysis within the fieldSegment function. Training samples were generated from a polygon layer consisting of 30 rectangular polygons for soil and 50 for plants, with the number of points per polygon set to 200.

### 2.6 Machine learning predictive models

The fitting of prediction models was performed according to Xing et al. (2018). All available FY observations at the plot level (630 plots × 5 harvests = 3,150 observations) were used as a prediction target to evaluate the predictive ability of five statistical models. The first two cuts were discarded due to very low values. The predictor variables included 19 RGB-derived indices and PH estimated from the DEM analysis. Prior to model fitting, the dataset was partitioned into two independent subsets using a group-based split approach. The plot-level dataset was divided into calibration (80%) and validation (20%) subsets based on unique alfalfa population names. To achieve this, random sampling without replacement was applied directly to the unique list of populations, assigning all corresponding phenotypic records (including replicates and harvests) of a specific entry exclusively to either the calibration or validation set. This grouping strategy resulted in a calibration dataset comprising 168 unique alfalfa populations (2,520 observations) and a validation dataset containing 42 unique populations (630 observations). The partitioning procedure was executed using the dplyr package in R software. Furthermore, predictor variables were preprocessed via centering, scaling, and the removal of highly correlated variables (*R* > 0.8 threshold) and those with near-zero variance.

Five predictive modeling ML approaches were evaluated: MLR, PLS, PCR, SVM, and ANN. Model calibration was performed using cross-validation based on the Venetian-blinds method with a 10-fold out-of-sample procedure. Model parameters were selected based on the root mean square error (RMSE) obtained during cross-validation. The PLS model was developed using four latent variables, whereas the PCR model was built using the first three principal components. The SVR model corresponded to an epsilon-SVR with a radial basis function (RBF) kernel. Its internal parameters were optimized as follows: cost = 3.1623, epsilon = 0.1, gamma = 0.031623, and 1,958 support vectors. Finally, the ANN model was implemented as a multilayer perceptron (MLP) consisting of one input node, two hidden-layer neurons, and one output node and was trained without prior dimensionality reduction of the predictor variables. Model predictive performance was evaluated using the coefficient of determination (*R*^2^) and the RMSE for both calibration and validation. All models were implemented in MATLAB version 9.14 (R2023a) (The MathWorks, Inc., 2023) using the PLS Toolbox (Eigenvector Research, Inc., 2023).

To statistically evaluate the differences in predictive accuracy between the baseline simple linear regression model (LM_PH_; FY = *a* + *b* × PH) and the five ML models (MLR, PLS, PCR, SVM, and ANN), a non-parametric Wilcoxon signed-rank test was conducted (Trawiński et al., 2012). The LM_PH_ model was evaluated using a 10-fold cross-validation framework, matching the procedure used for the complex ML models. The Wilcoxon test was performed on the independent validation dataset (*n* = 630) to execute a point-by-point comparison of prediction residuals. For each individual observation, the absolute prediction error (AE) was calculated as follows: AE = ∣FY_observed_ − FY_predicted_∣. The paired Wilcoxon test was utilized to contrast the AE vector of the LM_PH_ model against the corresponding AE vector of each ML approach. This non-parametric method obviates the need for normality assumptions. Statistical significance for all paired contrasts was established at a threshold of *P* < 0.05. The Wilcoxon signed-rank tests were performed using the native wilcox.test function in R (version 4.3.2).

### 2.7 Statistical analyses

In order to have an estimation of the broad-sense heritability (*H*^2^), an additional linear mixed model (LMM) was implemented to estimate variance components within the ASReml-R software (Butler et al., 2007) based on the following model:


$$y_{iljm}=\mu +R_{l}+h_{j}+bk_{lm}+g_{i}+hg_{ji}+e_{iljm}$$

where *y_ilmj_* is the phenotypic value of the *i*th genotype (*g*) in the *l*th replicate (R), *j*th harvest (h), and *lm*th incomplete block (bk); $h_{j}g_{i}$ is the harvest by genotype interaction; and μ is the overall population mean. For the best linear unbiased prediction (BLUP) estimation, the *R* was considered as a fixed effect, whereas all the other terms were considered as random, and e was the random experimental error assumed to be independent and with unique variance. The estimated variance components were used to estimate the *H*^2^ on a plot mean basis (Inostroza et al., 2018, 2019), which was calculated as follows:


$$H^{2}=\sigma_{g}^{2}/(\sigma_{g}^{2}+\sigma_{h}^{2}+\sigma_{bk}^{2}+\sigma_{hg}^{2}+\sigma_{e}^{2})$$

A complementary model was fitted that considered genotype as a fixed effect allowing to perform a pseudo-ANOVA test (Wald test) in ASReml-R software. The relationship among RGB indices and agronomical important traits (FY and PH) was studied with correlation analyses using the package corrplot in R software.

## 3 Results

### 3.1 Fall dormancy estimation and phenotypic variation

**Figure 1:**
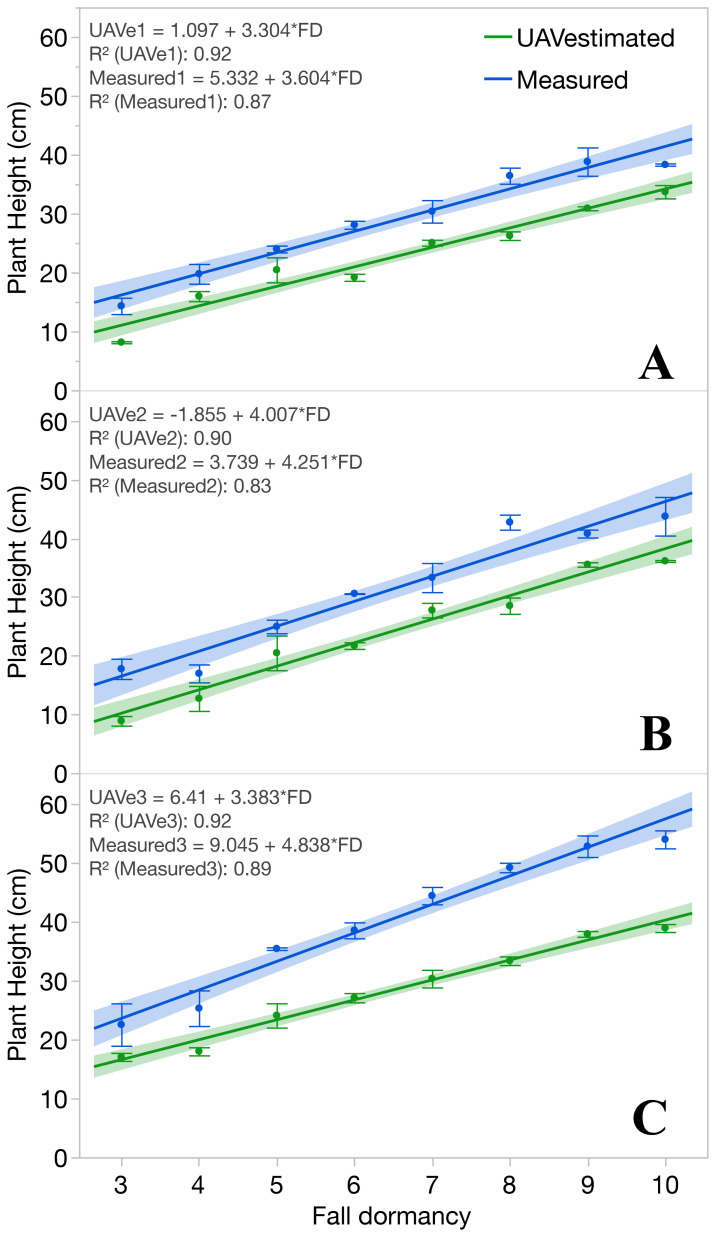
Relationship between plant height (PH), estimated by unmanned aerial vehicles (UAVestimated) and manual measurements (Measured), and fall dormancy (FD) of eight check cultivars (known FD score) evaluated at **(A)** 25, **(B)** 40, and **(C)** 60 days after the autumn equinox in 2025.

PH, measured manually in the autumn season, exhibited a strong and consistent linear relationship with FD scores of check cultivars (known FD score) across all three assessment dates (25, 40, and 60 days after the autumn equinox, DAEq). UAV-derived PH showed higher *R*^2^ values of 0.92, 0.90, and 0.92, respectively, compared to those obtained from manual measurements (*R*^2^ = 0.87, 0.83, and 0.89) (**Figure 1**). UAV-derived PH was consistently lower than manual measurements across dates, with reductions of 5.43%, 7.78%, and 3.26% at 25, 40, and 60 DAEq, respectively.

**Figure 2:**
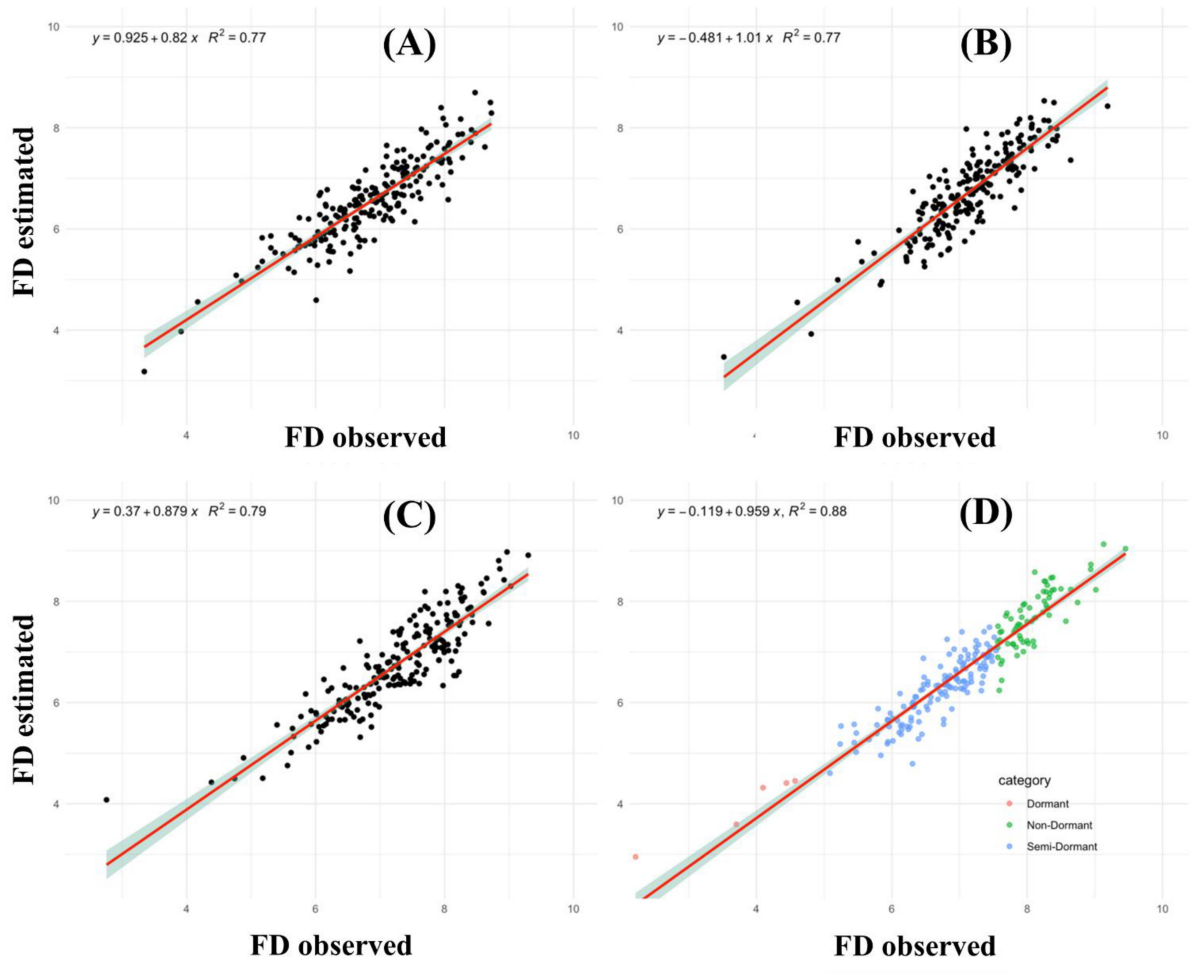
Relationship between UAV-estimated and manually observed fall dormancy **(FD)** scores for 210 alfalfa populations. Estimated and observed FD at **(A)** 25 days after the autumn equinox, **(B)** 40 days after the autumn equinox, **(C)** 60 days after the autumn equinox, and **(D)** across all three assessment dates in 2025.

Linear equations derived from the relationship between PH and FD scores of check cultivars (**Figure 1**) were used to estimate FD score of all 210 populations (FD = [(PH − *a*)/*b*]). Within each date of measurement, the intercept and slope of the linear equation varied slightly between UAV and manual estimated equations (**Figure 1**). The relationship between observed FD (manual PH evaluation) and UAV-estimated FD showed high consistency across the three measurement dates, with determination coefficients (*R*^2^) of 0.77, 0.77, and 0.79 at 25, 40, and 60 DAEq, respectively (**Figures 2A–C**). The linear equation estimated across all three assessment dates achieved an *R*^2^ value of 0.88 (**Figure 2D**). The FD values estimated across the three dates varied significantly among the alfalfa progenies (*P* < 0.001, **Table 1**). The 2%, 65%, and 33% of the progenies were categorized as dormant, semi-dormant, and non-dormant types, respectively (**Figure 2D**). Fall dormancy, estimated either by UAV or manually, exhibited an *H*^2^ value of 0.69 ± 0.04 and 0.64 ± 0.04, respectively (**Table 1**). These results demonstrate that canopy height extracted from UAV-based digital elevation models accurately captures the architectural differences associated with FD classes.

**Table 1:** Wald test statistics and broad-sense heritability (*H*^2^) for fall dormancy, plant height, and forage yield measured by UAV and manual methods in 210 alfalfa populations.

Phenotypic trait	Wald statistic			*H*^2^ value
	Population (*P*)	Harvest (*H*)	*P* × *H* interaction	
UAV-fall dormancy	2,460.1***	17.4***	124.8 ns	0.69 ± 0.04
Manual-fall dormancy	1,830.5***	42.8***	265.7 ns	0.64 ± 0.04
Forage yield	1,239.2***	6,253.2***	805.9 ns	0.33 ± 0.01
Plant height-UAV	1,387.5***	12,576.7***	599.8 ns	0.36 ± 0.02

***, Significant at P ≤ 0.001. ns, not significant.

### 3.2 Forage yield variability

**Figure 3:**
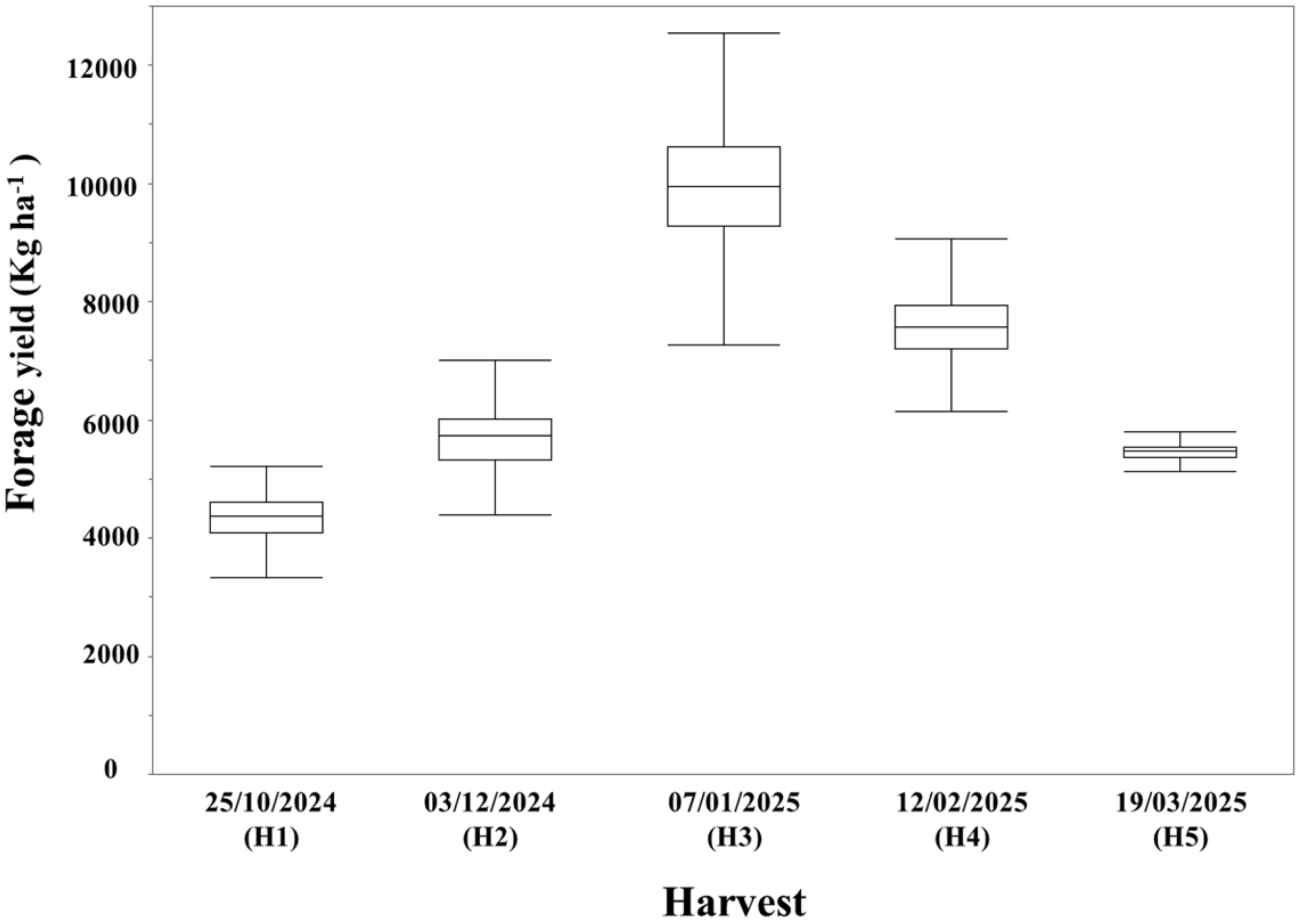
Boxplot of forage yield across harvests (H1 to H5) for 210 alfalfa populations grown in a Mediterranean environment in central Chile. Data are based on the best linear unbiased prediction (BLUP) value for each alfalfa population with three replications.

Seven harvests were performed across the two growing seasons (2022–2023 and 2024–2025). However, only five harvests were included in this analysis, as the first two harvests belonged to the establishment year. FY varied significantly among alfalfa populations (*P*) and harvest (*H*) (*P* < 0.001) but did not show a significant *P* × *H* interaction (*P* > 0.05; **Table 1**). FY increased from H1 and peaked at H3, followed by a progressive decrease at H4 and H5. FY across the 210 alfalfa populations ranged from 2,875 to 5,400 kg ha^−1^ for H1, 3,947 to 7,005 kg ha^−1^ for H2, 7,145 to 12,541 kg ha^−1^ for H3, 5,256 to 9,053 kg ha^−1^ for H4, and 4,959 to 5,821 kg ha^−1^ for H5 (**Figure 3**). The FY across all harvest exhibited an *H*^2^ value of 0.33 ± 0.01.

Significance levels: *P* ≤ 0.001***, *P* ≤ 0.01**, *P* ≤ 0.05*, and *P* > 0.05 = not significant (ns).

### 3.3 Forage yield, plant height, and UAV-derived indices

The PH and the 19 RGB VIs estimated across harvest with the UAV-based RGB imagery showed significant associations with FY. This indicates strong and significant effects of alfalfa populations and harvest (*P* < 0.001), with a non-significant population × harvest (*P* × *H*) interaction (**Table 1**; **Supplementary Table 1**).

**Figure 4:**
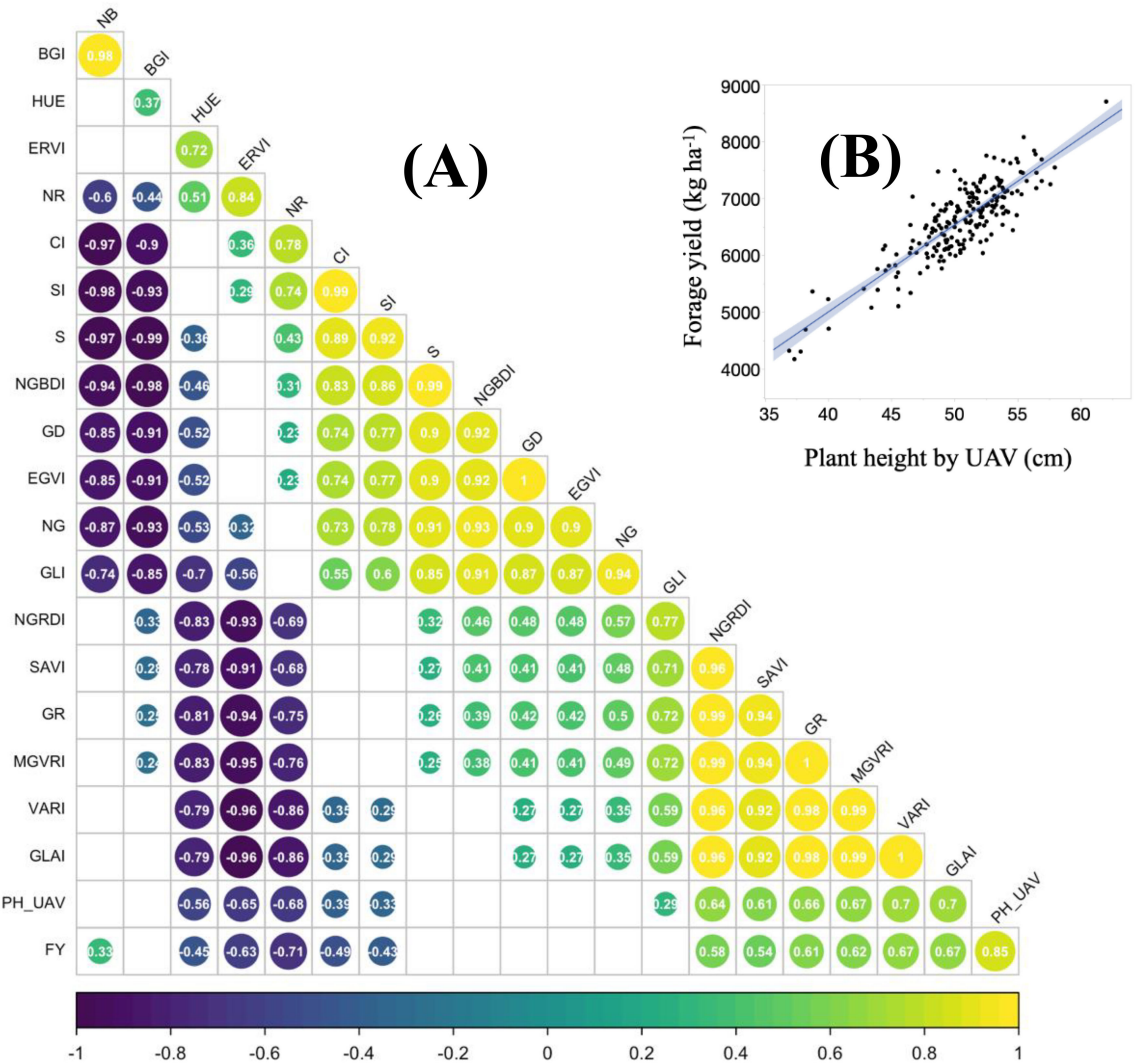
Pearson correlation coefficients among forage yield (FY) and RGB-derived vegetation indices **(A)**, and the relationship between plant height estimated with UAV methodology (PH_UAV) and FY **(B)** of 210 alfalfa populations. Color intensity represents the magnitude and direction of Pearson correlations. All correlation coefficients were significant at *P* < 0.001.

Pearson correlation among some VIs and FY revealed strong relationships (*P* < 0.001; **Figure 4A**). FY was negatively correlated with VIs, such as HUE, ERVI, NR, CI, and SI, with *r* values ranging from −0.43 to −0.71 (*P* < 0.001; **Figure 4A**). However, the Normalized Green-Red Difference Index (NGRDI), Soil-Adjusted Vegetation Index (SAVI), Green/Red Ratio (GR), Modified Green-Red Vegetation Index (MGVRI), Visible Atmospherically Resistant Index (VARI), Green Leaf Area Index (GLAI), and PH derived from the UAV-based methodology (PH-UAV) were positively correlated with FY with *r* values ranging from 0.54 to 0.85 (*P* < 0.001; **Figure 4A**). The VI NR showed a strong negative correlation (*R* = −0.71, *P* < 0.001) and GLAI and VARI showed a strong positive (*R* = 0.67, *P* < 0.001) correlation among all the indices with FY.

PH-UAV is particularly noteworthy, as it exhibited the strongest relationship with FY, with *r* and *R*^2^ values of 0.85 and 0.72, respectively (*P* < 0.001; **Figures 4A, B**). *H*^2^ of UAV-derived traits ranged from 0.01 to 0.58. The VIs ERVI and NR showed the highest *H*^2^ values (0.57 on average; **Supplementary Table 1**). The PH-UAV showed an *H*^2^ value of 0.36 ± 0.02 (**Table 1**).

### 3.4 Predictive ability of machine learning complex models

**Figure 5:**
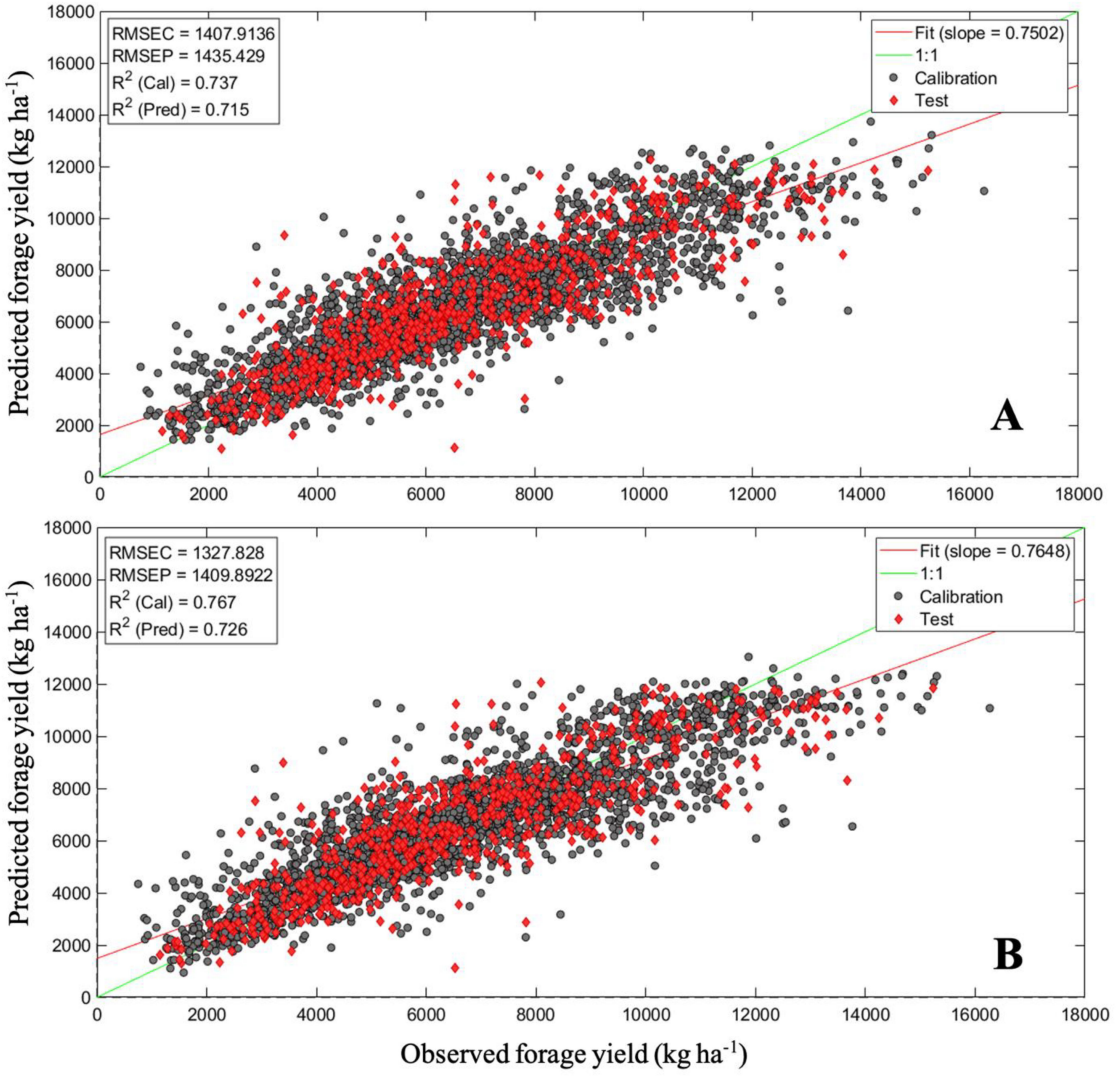
Prediction of forage yield for 210 alfalfa populations using the two best-fitting complex ML models. Black circles represent the calibration dataset, while red diamonds indicate the test dataset. The green line shows the 1:1 relationship between observed and predicted values, and the red line represents the fitted regression line. Models include **(A)** artificial neural network (ANN) and **(B)** support vector machine (SVM).

Machine learning models integrating PH-UAV and RGB-derived VIs significantly improved FY predictions relative to the baseline univariable simple linear regression model (LM_PH_) (**Table 2**, **Figure 5**). Except for PCR, all ML approaches significantly reduced absolute prediction errors compared to the LM_PH_ baseline (Wilcoxon V = 126,719 – 132,450, *P* < 0.001; **Table 2**).

**Table 2:** Summary of statistical fit for the univariable simple linear regression model with plant height as predictor (LM_PH_) and five complex multivariate models used to predict forage yield in 210 alfalfa populations grown in a Mediterranean environment in central Chile, across five harvests (2024–2025 growing season).

Model	Groups	*R*^2^cal	RMSEC (kg ha^-^¹)	*R*^2^val	RMSEP (kg ha^-^¹)	Wilcoxon *V*-value
LM_PH_	*n*_cal_ = 2,520 *n*_val_ = 630	0.63 ± 0.008	1,625.6 ± 23.3	0.65	1,301.7	
PLS	*n*_cal_ = 2,520 *n*_val_ = 630	0.66 ± 0.008	1,595.7 ± 22.5	0.68	1,514.8	126,719***
MLR	*n*_cal_ = 2,520 *n*_val_ = 630	0.73 ± 0.008	1,429.9 ± 23.2	0.70	1,459.7	127,180***
PCR	*n*_cal_ = 2,520 *n*_val_ = 630	0.54 ± 0.009	1,850.9 ± 24.3	0.57	1,752.1	86,453ns
ANN	*n*_cal_ = 2,520 *n*_val_ = 630	0.74 ± 0.007	1,407.9 ± 21.6	0.72	1,435.4	125,710***
SVM	*n*_cal_ = 2,520 *n*_val_ = 630	0.77 ± 0.007	1,327.8 ± 22.5	0.73	1,409.9	131,535***

Determination coefficient of the relationship between predicted and observed forage yield at calibration (*R*^2^cal) and validation (*R*^2^val) analyses. Number of observations in the calibration (*n*_cal_) and validation groups (*n*_val_). Significance levels: ***, significant at *P*<0.001. ns, not significant.

PLS, partial least squares; ANN, artificial neural network; SVM, support vector machine; MLR, multiple linear regression; PCR, principal component regression; RMSEC, root mean square error of calibration; RMSEP, root mean square error of prediction.

The ANN model achieved an *R*^2^ of 0.74 for calibration and an *R*^2^ of 0.72 for independent prediction. The model presented low bias and an RMSEP of 1,435.4 kg ha^−1^, indicating high predictive stability across datasets (**Figure 5A**). The MLR model achieved an *R*^2^ of 0.73 for calibration and an *R*^2^ of 0.70 for independent prediction with an RMSEP of 1,459.7 (**Table 2**). Among all the tested models, the PCR model, developed using three principal components derived from UAV indices, showed lower suitability due to its relatively high error values (RMSEC = 1,850.9, RMSEP = 1,752.1) and lower explanatory power (*R*^2^ = 0.54 for calibration and 0.57 for prediction) (**Table 2**). The PLS model showed lower performance, with *R*^2^ values of 0.66 (calibration) and 0.68 (prediction) and a higher RMSEP (1,514.8 kg ha^−1^). Although PLS handled multicollinearity, it was less capable of capturing complex relationships among traits. The SVM model demonstrated the best overall performance, with *R*^2^ values of 0.76 (calibration) and 0.73 (prediction), along with the lowest RMSEP (1,409.8 kg ha^−1^). Moreover, the slope of the fitted regression line (0.78) was closest to the 1:1 line, indicating relatively low prediction bias and good agreement between observed and predicted FY (**Table 2**, **Figure 5B**).

## 4 Discussion

In alfalfa breeding programs, two critical challenges are the objective estimation of FD and the accurate prediction of FY throughout the growing season. Therefore, novel phenotyping approaches are increasingly sought to support selection efficiency in breeding programs and improve the evaluation of these traits (Sapkota et al., 2023; Noushahi et al., 2025a). As explored in the present study, UAV-based RGB imagery presents a promising tool that could help bridge this gap.

### 4.1 UAV-based phenotyping accurately estimates alfalfa fall dormancy

The strong relationship between PH and FD across three measurement dates (25, 40, and 60 days after the autumn equinox) confirms previous findings that canopy architecture is intrinsically linked to alfalfa dormancy behavior (Cunningham et al., 1998; Ventroni et al., 2010; Liu et al., 2015). FDT genotypes typically reduce shoot elongation after the autumn equinox, whereas NDT cultivars tend to maintain active growth (Liu et al., 2019). Therefore, canopy height serves as a reliable proxy for FD expression. Previous studies have reported that alfalfa cultivars with higher FD ratings tend to develop longer internodes, whereas more dormant cultivars produce a greater number of internodes. There is a structural trade-off between internode length and internode number, with internode length identified as the primary trait contributing to differences in PH among alfalfa genotypes (Liu et al., 2015; Jing et al., 2024). The UAV-based approach successfully estimated PH and, consequently, FD based on the UPOV methodology. When genotypes were classified into the conventional biological categories (dormant, semi-dormant, and non-dormant), the UAV-estimated FD score maintained similar values to those obtained using conventional ruler-based measurements. In all three assessment dates, population assignments to FD categories were nearly identical. This demonstrates the robustness of HTP techniques. These findings are particularly important because it confirms that the UAV method does not merely correlate with high-labor scores (PH height measured with a ruler), but it also preserves the biological relevance and ordinal structure of FD classification. Furthermore, FD showed moderate to high *H*^2^ (0.64–0.69), which is coincident with other studies (Brummer et al., 2000; Li et al., 2015).

It is important to highlight that the manual FD assessment required two operators to measure PH over nearly 5 h. In contrast, the UAV-based methodology required approximately 10 min for flight acquisition and 1 h for image processing and data tabulation. Additionally, the UAV-estimated FD demonstrated superior statistical and genetic performance compared to manual measurements. For instance, the *R*^2^ for the relationship between PH and FD scores of check cultivars (**Figure 1**) was consistently higher using UAV-derived data than with manually acquired data. Similarly, the *H*^2^ values for FD estimated via the UAV methodology were higher than those from manual determination. This improved performance can be attributed to lower experimental noise in the UAV measurements (Pinto et al., 2023). On the other hand, manual measurements require the selection of representative plants, which may introduce sampling bias and measurement noise. Thus, the UAV methodology assesses a standardized section of the plot to determine total canopy height, providing a more robust and standardized approach.

These findings were consistent with previous studies, which demonstrate that remote sensing-based VIs can effectively and non-destructively estimate PH in alfalfa. It provides a scalable alternative to traditional visual FD scoring (Noland et al., 2018; Tang et al., 2021). From a practical breeding perspective, these findings are highly encouraging.

### 4.2 Phenotypic variation in alfalfa forage yield

Significant (*P* < 0.001) phenotypic variation was observed in FY across the five alfalfa harvests from 2023 to 2025 in the 210 alfalfa populations evaluated at the Santa Rosa Research Station (INIA Chile). This underscores the strong influence of seasonal environmental conditions and crop ontogeny on biomass production under Mediterranean conditions. The observed phenotypic variations were consistent with previous findings from alfalfa trials conducted in central Chile, particularly at Santa Rosa and Cauquenes, between 2017 and 2023 (Inostroza et al., 2021, 2026; del Pozo et al., 2023; Noushahi et al., 2025a, 2025b). The clear pattern of increasing FY from harvest 1 (H1) to a peak at H3 followed by a progressive decline in H4 and H5 is consistent with the growth dynamics of alfalfa in Mediterranean climates (Djaman et al., 2021).

Early harvests (H1 and H2 correspond to late winter and spring, respectively) generally reflect establishment-phase limitations, including lower root development (Petcu et al., 2019), recovery from winter (Pembleton et al., 2011), incomplete canopy closure, less solar radiation interception (Jia et al., 2022), and ultimately lower photosynthetic capacity resulting in narrower FY ranges. The pronounced peak at H3 (summer) (7,145 to 12,541 kg ha^−1^) is a consequence of the optimal environmental conditions (Noushahi et al., 2025a) combined with the fully developed root system and maximum leaf area index. Together, these traits could maximize light interception, carbon assimilation, and ultimately biomass accumulation (Pan et al., 2023; Zhang and Wang, 2025; Noushahi and del Pozo, 2026). Moreover, this harvest captured the wide range of FY variation (5,396 kg ha^−1^). This suggests that summer conditions provided the greatest expression of yield potential and adaptation among the evaluated alfalfa populations. The decline in FY during H4 and H5 (early autumn) reflects the combined effects of shortening day length, shorter interval between harvests, cooler temperatures, reduced photosynthetic efficiency, and the onset of FD induction (Xu and Min, 2022). During this period, genotypes may begin preparing for dormancy by translocating photosynthates from the canopy to the crown root reserves for winter survival, which progressively restricts regrowth and canopy development (Noushahi and del Pozo, 2026).

### 4.3 UAV-based derived indices correlated with forage yield

RGB-derived indices showed clear directional associations with alfalfa FY. These indices reflect their sensitivity to canopy greenness, vigor, and structural traits (**Supplementary Table 1**). Indices such as GLAI, VARI, MGVRI, GR, SAVI, NGRDI, BGI, NB, and Green Leaf Index (GLI) exhibited strong positive correlations with FY. Several RGB-based VIs used in this study have been widely documented in previous research. For example, SAVI reduces the influence of soil brightness on vegetation signals (Huete, 1988). Similarly, VARI is designed to minimize atmospheric effects and can estimate vegetation fraction with an error of less than 10% across a wide range of atmospheric conditions (Gitelson et al., 2002). Other indices such as the GLI (Louhaichi et al., 2001) and GLAI (Nguy-Robertson et al., 2014) also emphasize canopy greenness and chlorophyll-related reflectance. Collectively, these indices enhance vegetation signals while reducing background noise. This suggests that genotypes with denser, greener, and more vigorous canopies consistently produced higher FY.

On the other hand, indices such as NR, ERVI, HUE, SI, and CI displayed strong negative correlations with both traits. Previous studies confirm that these indices often highlight red or HUE-based components such as the Coloration Index (CI) (Inostroza et al., 2026). In alfalfa, it refers to the concentration of anthocyanins, which are flavonoids that contribute to the color of plant canopy (Cao et al., 2025). Studies also support our findings that the negative relationship between CI-related indices and FY may result from spectral signal saturation at high canopy density (Huete, 2012; Yaschenko et al., 2025). The RGB indices NR and ERVI showed higher *H*^2^ values (*H*^2^ = 0.57; **Supplementary Table 1**) and strong correlation with FY (**Figure 2A**), making them suitable selection criteria.

Among all UAV-derived traits, PH showed the strongest relationship with alfalfa FY (*R*^2^ = 0.72). Similar findings have been reported in previous studies; for example, a 2-year trial of 10 alfalfa varieties demonstrated a positive correlation between PH and FY, which supports its use as a yield indicator (Jia et al., 2022). Likewise, another study showed that alfalfa yield decreased with increasing cutting height (Vuković et al., 2025). This strong association indicates that UAV-based PH measurements could serve as a practical proxy for estimating FY. It could reduce the need for destructive and labor-intensive harvesting across the entire experimental area.

### 4.4 Machine learning improves forage yield prediction

FY is driven by complex interactions among canopy traits, and ML models, particularly SVM and ANN, effectively captured these complex relationships. The reason is that ML algorithms provide higher predictive accuracy than conventional linear multivariate approaches (Castillo-Girones et al., 2025; Fu et al., 2025). A study evaluated satellite remote sensing bands, VIs, and ML models to accurately predict alfalfa FY during different harvest times in 2022. Among the tested ML models, the SVM model using multiple parameters provided higher prediction accuracy (*R*^2^ ≈ 0.65) than single-parameter models (Li et al., 2023). In addition, Li et al. (2023) reported good *R*^2^ ≈ 0.50 for the ANN model just like in our study (*R*^2^ = 0.72), but its RMSE and bias were high compared to our study. This may be due to a smaller sample size and poor training. In contrast, our study showed lower RMSEP of 1,435.4 kg ha^−1^ for ANN among ML models, indicating improved model training with a larger dataset (An et al., 2024). A previous study used ANN to predict the impact of climate change on alfalfa yield in Turkey. Using cultivation data, soil parameters, and climate data, the best ANN model (from 176 alternatives) predicted century yields with good accuracy (*R*^2^ = 0.83) (Pekin et al., 2023). SVM outperformed PLS for all variables by providing more accurate estimates of FY and supporting harvest management decisions when both were compared in a study conducted in Sweden using canopy spectral reflectance (400 to 1,000 nm) from 377 grass legume mixture samples (Zhou et al., 2019). Although PLS performed well, it did not perform as well as SVM in our study, which also confirmed similar findings reported in another research. As shown in **Figure 5**, while the SVM model achieved high predictive accuracy (*R*^2^ = 0.73), the slope of the regression line (0.76) indicates a slight bias, where the model tends to marginally underestimate the highest-yielding populations and overestimate the lowest. This compression of the predicted range is a common characteristic of ML models in biological systems and should be considered when applying these models for extreme trait selection (Feng et al., 2020). The PCR model was ineffective in our study, indicating its limited suitability for predicting alfalfa FY and probably explaining its limited use for FY prediction in the literature. However, a study using hyperspectral remote sensing (350 to 1,050 nm) and ML to estimate mango leaf nutrients also reported that PCR was ineffective (Mahajan et al., 2021). The SVM and ANN models achieved significantly higher predictive performance than the baseline LM_PH_ model (*P* < 0.001; **Table 2**). Although the ML models yielded a modest numerical increase in *R*^2^ values compared to the LM_PH_, their implementation carries crucial implications for applied alfalfa breeding. In large-scale breeding programs where hundreds or thousands of candidate lines are evaluated simultaneously, even incremental gains in predictive accuracy can substantially enhance selection intensity and genetic gain (Annicchiarico, 2021, b). Furthermore, because the data acquisition and computational pipelines for both the LM_PH_ and ML approaches are virtually identical, deploying these advanced models provides a more robust, multitrait integration of plant architecture and physiological performance without incurring any additional labor or financial costs.

Remote sensing using RGB imagery effectively estimates alfalfa FY by capturing key physiological traits that regulate growth (Hammond et al., 2023). Reflectance in the visible spectrum, specifically in the red and green bands, is strongly influenced by chlorophyll absorption and leaf cellular structure (Garriga et al., 2020; Hammond et al., 2023). Consequently, RGB-derived vegetation indices, such as MGRVI, NGRDI, and EGVI, serve as indirect estimators of chlorophyll content and leaf area index (LAI), both of which are closely linked to photosynthetic capacity and biomass production (Hammond et al., 2023). LAI is a critical determinant of biomass accumulation in alfalfa, because it regulates the interception of photosynthetically active radiation. However, this relationship exhibits a saturation threshold (Liu et al., 2021). Furthermore, structural parameters like canopy height indicate the allocation of assimilates to physical growth, offering information that complements spectral indices. The superior performance of complex models, such as ANN and SVM over linear approaches, is rooted in these complex, multidimensional physiological dynamics. While linear models often assume constant relationships, they fail to account for the LAI saturation threshold where spectral signals become less responsive despite continued increases in plant biomass. In contrast, complex algorithms effectively capture the synergy between biochemical indicators and structural traits. Consequently, the improved accuracy observed in our results reflects the ability of these models to capture the complex interactions between canopy traits and radiation interception efficiency.

### 4.5 Study limitations and future directions

Although UAV-based indices successfully estimated plant height, fall dormancy, and forage yield in alfalfa, this study acknowledges certain technological limitations. For instance, our research relied solely on RGB imagery, which is restricted to three spectral bands primarily associated with photosynthetic status (chlorophyll; **Supplementary Table 1**). To address this, our ongoing work incorporates multispectral and hyperspectral cameras, as well as LiDAR technology, which we will expect to enhance the predictive power of multivariate models. Nevertheless, these advanced technologies remain expensive for large-scale plant breeding schemes, which is why RGB technology was prioritized. At the current global stage, RGB remains the most accessible and cost-effective tool for breeders worldwide.

Furthermore, while this dataset is robust, which was comprising 630 experimental units evaluated across two growing seasons and multiple harvests, it was limited to a single location. Consequently, the genotype × environment (G × E) interactions for RGB-derived traits have not yet been fully explored. To overcome this limitation, we have established an additional experiment in Cauquenes, a site prone to severe drought. This will allow us to evaluate more diverse environmental effects, accounting for different soil types and varying patterns of rainfall amount and annual distribution.

## 5 Conclusion

UAV-derived plant height and fall dormancy showed strong linear relationships with manual measurements across three dates. This indicates that UAV-derived traits can serve as reliable proxies for estimating PH and FD in alfalfa breeding programs. Forage yield was positively associated with RGB-derived indices such as GLAI, VARI, VIG, MGVRI, GR, SAVI, NGRDI, NB, and GLI and negatively associated with NR, ERVI, HUE, SI, and CI. The most effective predictor for FY was UAV-estimated PH (*R*^2^ = 0.72) and can serve as a direct proxy during the early stages of breeding programs when large numbers of plots are evaluated simultaneously. Machine learning models integrating PH and RGB-derived vegetation indices significantly improved FY prediction, with *R*^2^ increasing from 0.65 (LM_PH_) to 0.73 using SVM. Among the evaluated models, SVM and ANN most effectively captured the underlying complex relationships. The high-throughput phenotyping tools implemented in this study proved to be reliable, labor-efficient, rapid, and non-destructive. These tools provide a valuable prospective framework for evaluating alfalfa populations and may offer insights into alternative strategies for optimizing genetic gain in this genetically complex species.

## Data Availability

The original contributions presented in the study are included in the article/supplementary material. Further inquiries can be directed to the corresponding author.
