# Supplementary material for: UAV image-derived canopy traits for predicting alfalfa fall dormancy and forage yield in Mediterranean environments
Source: Front Plant Sci. 2026 Jul 17;17:1841672. doi: 10.3389/fpls.2026.1841672 (PMC13425762; doi:10.3389/fpls.2026.1841672)
Supplement: Supplementary file 1 [file Table1.docx]

**Supplementary Table 1.** Description, Wald test, and broad sense heritability (H^2^) of 19 RGB derived indices estimated in 210 alfalfa populations established in a Mediterranean environment in central Chile.

| **Vegetation Index** | **Description** | **Formula** | **Related traits** | **Wald statistic** | | | **H^2^ value** |
| --- | --- | --- | --- | --- | --- | --- | --- |
|  |  |  |  | **Population (P)** | **Harvest (H)** | **PxH interaction** |  |
| BGI | Blue Green Pigment Index | B/G | Chlorophyll, LAI | 678.1*** | 6285.4*** | 481.3 ns | 0.21±0.10 |
| CI | Coloration Index | ((R-B)/R) | Soil color | 998.6*** | 6259.8*** | 591.2 ns | 0.31±0.13 |
| EGVI | Excess Green Vegetation Index | 2*G-R-B | Weed identification | 543.0*** | 19359.8*** | 421.7 ns | 0.07±0.04 |
| ERVI | Excess Red Vegetation Index | ((1.4*R)-G) | Vegetation identification | 1212.0*** | 1270.7*** | 718.3 ns | 0.58±0.08 |
| GD | Green Difference | G-(R+B)/2 | Vegetation monitoring | 543.0*** | 19359.8*** | 421.7 ns | 0.07±0.04 |
| GLAI | Green Leaf Area Index | (25*(G-R)/(G+R-B)+1.25) | Leaf area | 1118.9*** | 2827.5*** | 536.6 ns | 0.45±0.11 |
| GLI | Green Leaf Index | (2*G-R-B)/(2*G+R+B) | Chlorophyll | 648.9*** | 6001.3*** | 432.0 ns | 0.21±0.10 |
| GR | Green-Red Index | G/R | Vegetation monitoring | 1040.4*** | 3640.1*** | 559.5 ns | 0.40±0.12 |
| MGVRI | Modified Green Vegetation Ratio Index | (G^2-R^2)/(G^2+R^2) | Biomass monitoring | 1000.9*** | 3581.1*** | 496.2 ns | 0.39±0.12 |
| NB | Normalized Blue | B/(R+G+B) | Early nitrogen requirements | 802.7*** | 6671.2*** | 517.7 ns | 0.24±0.12 |
| NG | Normalized Green | G/(R+G+B) | Early nitrogen requirements | 308.4*** | 28289.7*** | 372.6 ns | 0.02±0.01 |
| NGBDI | Normalized Green-Blue Difference Index | (G-B)/(G+B) | Vegetation extraction | 645*** | 35494*** | 664 ns | 0.05±0.03 |
| NGRDI | Normalized Green Red Difference Index | (G-R)/(G+R) | Chlorophyll, biomass, water content | 1058*** | 253791*** | 681 ns | 0.01±0.01 |
| NR | Normalized Red | R/(R+G+B) | Early nitrogen requirements | 1609*** | 2955*** | 732 ns | 0.56±0.12 |
| HUE | Overall Hue Index | atan(2*(B-G-R)/30.5*(G-R)) | Soil color | 400*** | 573*** | 604 ns | 0.34±0.07 |
| S | Saturation | ((R+G+B)-3*B)/(R+G+B) | Crop growth estimation | 597*** | 24339.8*** | 708.4 ns | 0.06±0.04 |
| SAVI | Soil Adjusted Vegetation Index | (1+0.5)*(G-R)/(G+R+0.5) | Soil-adjusted vegetation | 385*** | 42162*** | 624 ns | 0.02±0.01 |
| SI | Spectral Slope Saturation Index | (R-B)/(R+B) | Soil color | 847.7*** | 5449.0*** | 521.3 ns | 0.29±0.13 |
| VARI | Visible Atmospherically Resistant Index | (G-R)/(G+R-B) | Canopy, biomass, chlorophyll | 1118.9*** | 2827.5*** | 536.6 ns | 0.45±0.11 |

Significance levels: *p* ≤ 0.001***, *p* ≤ 0.01***, p ≤* 0.05* and p > 0.05 = not significant (ns).
